# Myocardial perfusion using cardiac magnetic resonance: Evidence of decreased global myocardial perfusion in patients with systemic sclerosis with possible gender differences

**DOI:** 10.1186/1532-429X-18-S1-P71

**Published:** 2016-01-27

**Authors:** Tom Gyllenhammar, Mikael Kanski, Henrik Engblom, Dirk M Wuttge, Marcus Carlsson, Roger Hesselstrand, Håkan Arheden

**Affiliations:** 1 Cardiac MR Group Dept of Clinical Physiology, Department of Clinical Sciences, Lund, Sweden; 2 Section of Rheumatology, Department of Clinical Sciences, Lund, Sweden

## Background

Significant cardiovascular mortality is reported in patients with systemic sclerosis (SSc) despite that there is moderate to no increase of signs of epicardial coronary artery disease. This could be due to reduced myocardial perfusion caused by microvascular disease. The quantitative extent of hypoperfusion in patients with SSc, however, is not known. Recently, general interest has emerged around possible gender influence in various cardiac pathologies and hence we performed a gender adjusted comparison in this study. The purpose of this study was therefore to quantitatively measure if patients with SSc have decreased global myocardial perfusion (MP) at rest and during adenosine stress in a gender adjusted cohort.

## Methods

Cardiovascular magnetic resonance imaging (CMR) was conducted in 17 SSc patients (15 females, 45-74 years) and 11 controls (6 females, 44-66 years). Twelve patients had limited SSc and five patients had diffuse cutaneous SSc. One patient had pulmonary arterial hypertension. MP was quantified using coronary sinus flow at rest and adenosine stress, divided by left ventricular mass. Myocardial fibrosis was assessed using late gadolinium enhancement (LGE).

Cardiovascular magnetic resonance imaging (CMR) was conducted in 17 SSc patients (15 females, 45-74 years) and 11 controls (6 females, 44-66 years). Twelve patients had limited SSc and five patients had diffuse cutaneous SSc. One patient had pulmonary arterial hypertension. MP was quantified using coronary sinus flow at rest and adenosine stress, divided by left ventricular mass. Myocardial fibrosis was assessed using late gadolinium enhancement (LGE).

## Results

**Figure 1:**
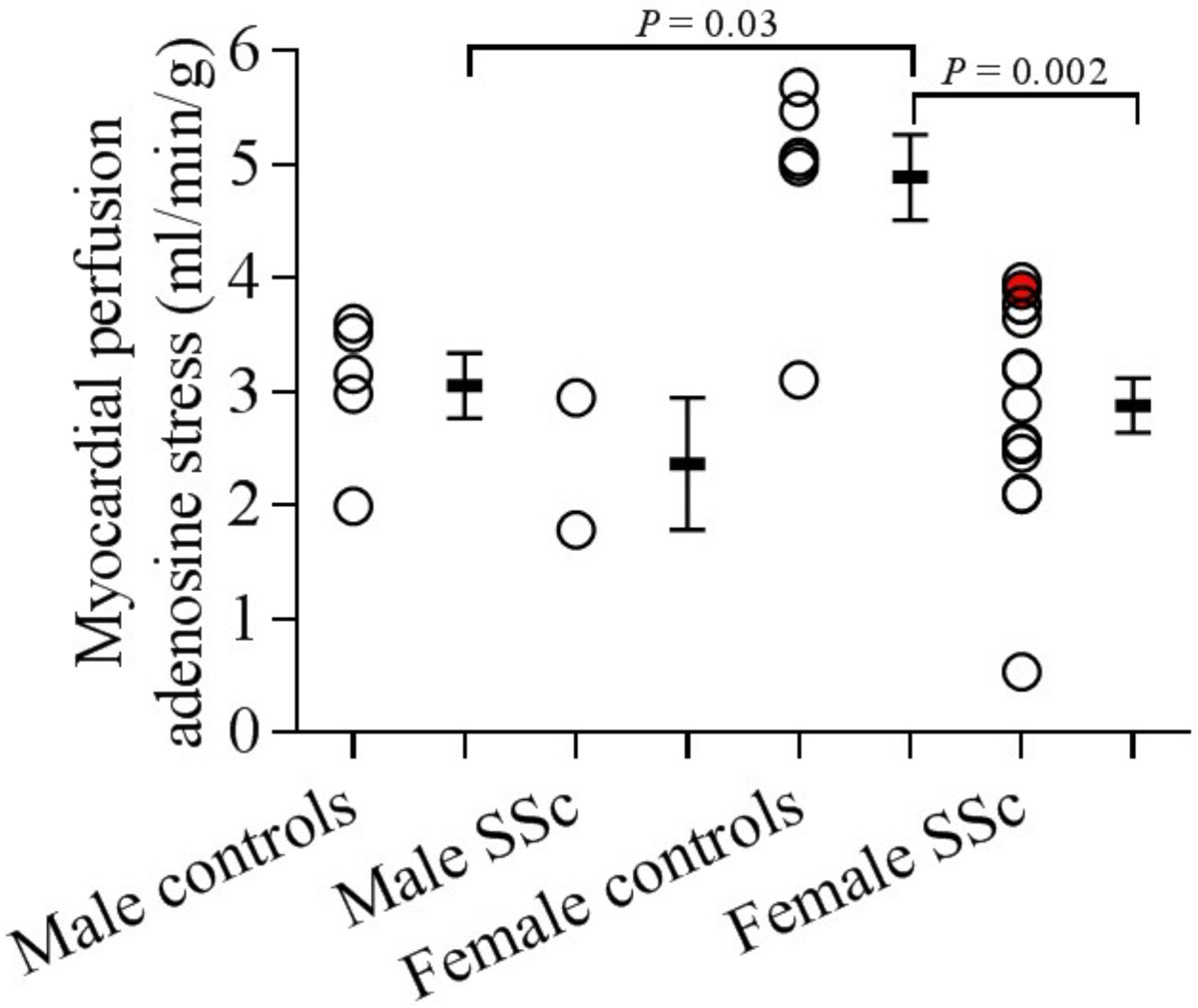
**Myocardial perfusion (MP) at adenosine stress**. Patients and controls are split into subgroups based on gender. Female SSc patients have significantly lower MP compared to female controls, but not compared to male controls. Statistical comparisons with male SSc patients were not performed due to the small study population. PAH patient marked with solid red circle. Error bars show mean ± SEM.

MP were similar at rest in patients and controls (1.2 ± 0.2 vs. 1.1 ± 0.1 ml/min/g, *P* = 0.85) whereas patients with SSc showed significantly lower MP during adenosine stress (2.8 ± 0.2 vs. 4.1 ± 0.4 ml/min/g, *P* = 0.025). There was significantly lower MP during adenosine stress in female SSc patients compared to female controls (2.9 ± 0.2 vs. 4.9 ± 0.2 ml/min/g, *P* = 0.002), fig 1. In patients, MP was within the same range in both men and women. Healthy men, however, have a significantly lower MP during adenosine stress than healthy women (3.1 ± 0.3 vs. 4.9 ± 0.4 ml/min/g, *P* = 0.03). No patient had signs of myocardial infarction.

## Conclusions

Global MP is decreased during adenosine stress, but not at rest, in patients with SSc compared to healthy controls and preliminary data suggest that there may be a difference between women and men. Gender adjustment may therefore be important when investigating MP. The CMR method using coronary sinus flow to measure global myocardial perfusion may be a candidate to mark myocardial microvascular disease in SSc patients.

